# CCL3 promotes angiogenesis by dysregulation of miR-374b/ VEGF-A axis in human osteosarcoma cells

**DOI:** 10.18632/oncotarget.6708

**Published:** 2015-12-21

**Authors:** Yuan-Ya Liao, Hsiao-Chi Tsai, Pei-Yu Chou, Shih-Wei Wang, Hsien-Te Chen, Yu-Min Lin, I-Ping Chiang, Tzu-Ming Chang, Shao-Keh Hsu, Ming-Chih Chou, Chih-Hsin Tang, Yi-Chin Fong

**Affiliations:** ^1^ Institute of Medicine, Chung Shan Medical University, Taichung, Taiwan; ^2^ Department of Surgery, Chung Shan Medical University Hospital, Taichung, Taiwan; ^3^ Graduate Institute of Basic Medical Science, China Medical University, Taichung, Taiwan; ^4^ Department of Nursing, Hung Kuang University, Taichung, Taiwan; ^5^ Department of Medicine, Mackay Medical College, New Taipei City, Taiwan; ^6^ Department of Orthopedic Surgery, China Medical University Hospital, Taichung, Taiwan; ^7^ School of Chinese Medicine, China Medical University, Taichung, Taiwan; ^8^ Department of Medicine, Chung Shan Medical University, Taichung, Taiwan; ^9^ Department of Orthopaedics, Taichung Veterans General Hospital, Taichung, Taiwan; ^10^ Department of Pathology, China Medical University Hospital, Taichung, Taiwan; ^11^ Department of Orthopedic Surgery, Tungs' Taichung Metroharbor Hospital, Taichung, Taiwan; ^12^ Department of Pharmacology, School of Medicine, China Medical University, Taichung, Taiwan; ^13^ Department of Biotechnology, College of Health Science, Asia University, Taichung, Taiwan; ^14^ Department of Sports Medicine, College of Health Care, China Medical University, Taichung, Taiwan

**Keywords:** MAPK, miR-374b, VEGF-A, angiogenesis, osteosarcoma

## Abstract

Osteosarcoma is the most frequent bone tumor, characterized by a high metastatic potential. However, the crosstalk between chemokine (C-C motif) ligand 3 (CCL3), which facilitates tumor progression and metastasis. Vascular endothelial growth factor-A (VEGF-A), an angiogenesis inducer and a highly specific mitogen for endothelial cells, has not been well explored in human osteosarcoma. Here we demonstrate the correlation of CCL3 and VEGF-A expressions, quantified by immunohistochemistry, with the tumor stage of human osteosarcoma tissues. Furthermore, CCL3 promotes VEGF-A expression in human osteosarcoma cells that subsequently induces human endothelial progenitor cell (EPC) migration and tube formation. Phosphorylation of JNK, ERK, and p38 was found after CCL3 stimulation. In addition, JNK, ERK, and p38 inhibitors also abolished CCL3-induced VEGF-A expression and angiogenesis. We noted that CCL3 reduces the expression of miR-374b and miR-374b mimic by reversing CCL3-promoted VEGF-A expression and angiogenesis *in vitro* and *in vivo*. This study shows that CCL3 promotes VEGF-A expression and angiogenesis in human osteosarcoma cells by down-regulating miR-374b expression via JNK, ERK, and p38 signaling pathways. Thus, CCL3 may be a new molecular therapeutic target in osteosarcoma angiogenesis and metastasis.

## INTRODUCTION

Angiogenesis is a physiological process through which new blood vessels form from pre-existing vessels. It is involved in the growth, maintenance, and metastasis of most of the solid tumors [1, 2]. Osteosarcoma is the most common primary malignant bone tumor. Today, the use of chemotherapy has remarkably augmented the survival rates; patients have about 65-70% chance of 5-year relapse-free survival, if the disease is localized [3]. However, patients with pulmonary metastasis of osteosarcoma have a very poor prognosis with a relapse within 5 years [4, 5]. It is known that vascular endothelial growth factor-A (VEGF-A) is one of the major inducers of angiogenesis [6] and plays an important role in the pathogenesis and progression of various cancers [7]. Our previous study reported that VEGF-A expression is associated with the osteosarcoma clinical stage [8]. Therefore, it is necessary to understand the effect of VEGF-A in angiogenesis pathways and its mechanism in human osteosarcoma.

Environmental factors such as growth factors, cytokines, and chemokines stimulate cancer cells to secrete VEGF-A [9]. Chemokine (C-C motif) ligand 3 (CCL3), also known as macrophage inflammatory protein-1α (MIP-1α), belongs to the family of chemokines [10]. CCL3 and its receptors, CCR1 and CCR5, contribute to the development of bone disease in multiple myeloma by supporting tumor growth and regulating osteoclast differentiation [11]. CCL3 is also associated with the regulation of cell growth, angiogenesis, and metastasis of different tumors such as melanoma [12], renal cell carcinoma [13], and colorectal cancer [14]. Moreover, CCL3 enhances cell migration and metastasis by up-regulating matrix metalloproteinase-2 (MMP)-2 expression in chondrosarcoma cells [15].

Several studies have focused on the role of microRNAs (miRNAs) in cancer progression [16]. MiRNAs influence numerous cancer-relevant processes such as proliferation, apoptosis, migration, invasion, and angiogenesis [17]. MiRNAs are short noncoding RNA molecules, with an average length of about 18 to 22 nucleotides. They bind to the 3′ untranslated region (3′-UTR) of mRNA through complementary base pairing, resulting in mRNA degradation or translation inhibition [18]. Previous studies have shown that miRNAs inhibit tumor angiogenesis through dysregulation of miR/VEGF-A axis [19-22]. MicroRNA-374b (miR-374b) promotes gastric cancer cell invasion and metastasis by suppressing RECK expression [23]. Overexpression of miR-374b mimic attenuates the mRNA level of VEGF-A in PC-3 prostate cancer cells [24]. However, the role of miR-374b in human osteosarcoma is largely unknown.

Development of an anti-angiogenic and anti-metastatic therapy can be useful for patients with poor prognosis of osteosarcoma distant metastasis [25]. CCL3 promotes metastasis in several cancers [26-28]. However, the role of CCL3 in VEGF-A production and angiogenesis in human osteosarcoma cells has not yet been clarified. Here we show that CCL3 enhances tumor angiogenesis and VEGF-A expression by dysregulation of miR-374b via the JNK, ERK, and p38 pathways in human osteosarcoma.

## RESULTS

### CCL3 regulates angiogenesis by increasing VEGF-A expression in human osteosarcoma cells

**Figure 1:**
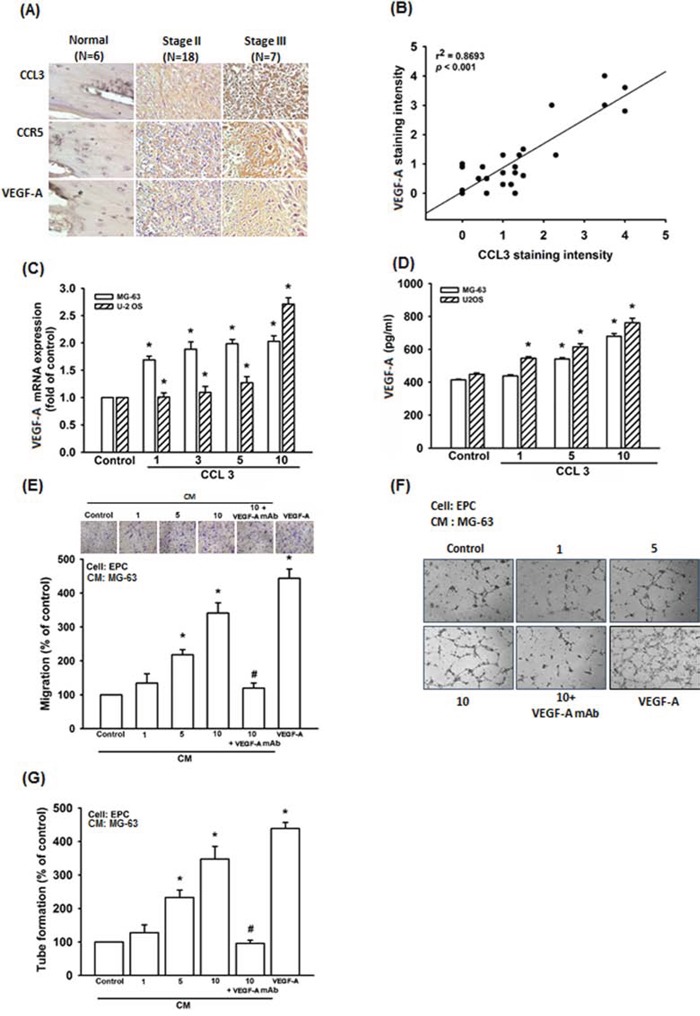
CCL3 enhances angiogenesis by increasing VEGF-A expression in human osteosarcoma cells **A.** Thirty-one tumor specimens were immunostained (IHC) with anti-CCL3, CCR5 and VEGF-A antibody. **B.** Quantitative results and correlation between CCL3 and VEGF-A clinical grade. **C&D.** Cells were treated with various concentration of CCL3, and the mRNA and protein expression were detected by RT-qPCR and ELISA. **E&F.** Cells were pre-treated for 30 min with VEGF-A antibody (20 ng/mL), followed by stimulation with CCL3 (1-10 ng/mL) for 24 h. The culture medium was collected as CM and then applied to EPCs for 24 h. The cell migration and capillary-like structure formation in EPCs was examined by Transwell and tube formation assay. **G.** Quantitative results of EPCs tube formation assay. Each experiment was done in triplicate. Results are expressed as mean ± S.E.M. *P<0.05 compared with control; ^#^*P*<0.05 compared with CCL3-treated group.

CCL3 promotes cancer metastasis and progression in lung cancer, oral squamous carcinoma, and chondrosarcoma cells [13, 15, 28]. However, the role of CCL3 in human osteosarcoma cell is mostly unknown. The immunohistochemistry (IHC) result indicated that CCL3 expression is higher in osteosarcoma patients compared to normal individuals, and the expression profile of CCL3 is associated with the osteosarcoma clinical stage (Fig. 1A). According to our previous report, VEGF-A expression is also associated with osteosarcoma clinical stage [8]. Therefore, we analyzed the expression profiles of CCL3 and VEGF-A, and found that the expression of CCL3 has positive correlation with VEGF-A (Fig. 1B). We treated osteosarcoma cells with various concentrations of CCL3 to determine the relationship between CCL3 and VEGF-A. As results, CCL3 stimulation promoted VEGF-A mRNA expression at 2-2.5 fold and protein expression from 414-448 to 680-762 (pg/ml) (Fig 1C and 1D). These data demonstrated that CCL3 enhanced VEGF-A expression in osteosarcoma cells. Angiogenesis involves proliferation, migration, and tube formation of endothelial progenitor cells (EPCs) to form new blood vessels [29]. We found that the conditioned medium (CM) from CCL3-treated osteosarcoma cells promoted EPCs migration and tube formation (VEGF-A was used as positive control) (Fig. 1E-1G and Supplementary Fig. S1). In addition, pretreatment with a VEGF-A neutralizing antibody abolished these effect (Fig. 1E-1G and Supplementary Fig. S1). Hence, CCL3 promotes angiogenesis through VEGF-A-dependent expression in human osteosarcoma cells.

### Knockdown of CCL3 decreases VEGF-A expression and angiogenesis in human osteosarcoma cells

**Figure 2:**
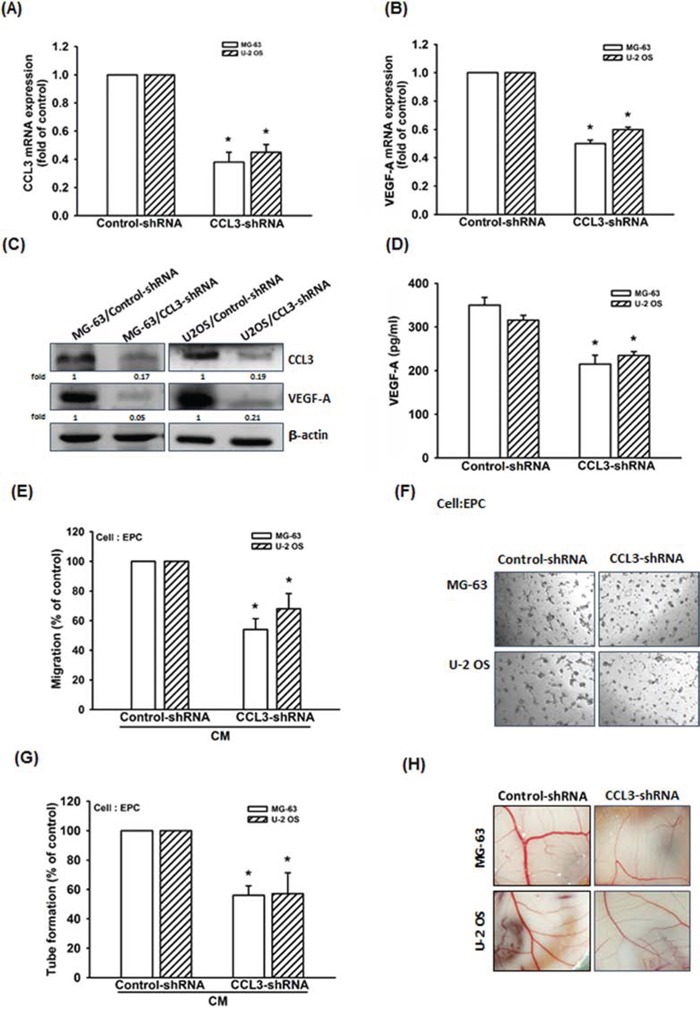
Knockdown of CCL3 decreases VEGF-A expression and inhibits angiogenesis in human osteosarcoma cells **A-D.** The mRNA and protein expression of CCL3 and VEGF-A in Control- or CCL3-shRNA osteosarcoma cells were detected by RT-qPCR, western blot, and ELISA. **E&F.** The culture medium were collected as CM and then applied to EPCs for 24 h. The cell migration and capillary-like structure formation in EPCs was examined by Transwell and tube formation assay. **G.** Quantitative results of EPC tube formation assay. **H.** Chick embryos were incubated with osteosarcoma CM for 4 days, and then photographed with a stereomicroscope. Each experiment was done in triplicate. Results are expressed as mean ± S.E.M. *P<0.05 compared with control.

To further confirm CCL3-mediated VEGF-A expression and angiogenesis in human osteosarcoma, we constructed MG-63 and U-2 OS cells stably expressing CCL3-shRNA. The mRNA and protein expressions of CCL3 decreased in MG-63/CCL3-shRNA and U-2 OS/CCL3-shRNA cells compared to control cells, as shown in Fig. 2A and 2C. Furthermore, knockdown of CCL3 decreased the mRNA and protein expressions of VEGF-A (Fig. 2B-2D), but did not affect the cell viability in these cells (Supplementary Fig. S2). We also examined the angiogenic function of the recruited EPCs, and the data indicated that CM collected from MG-63/CCL3-shRNA or U-2 OS/CCL3-shRNA cells inhibited EPC migration and tube formation (Fig. 2E-2G). Besides, we also used chick chorioallantoic membrane (CAM) model to detect the angiogenesis. We found that the angiogenesis was inhibited in MG-63/CCL3-shRNA and U-2 OS/CCL3-shRNA cells (Fig. 2H). Taken together, these data indicating that CCL3 plays an important role in promoting VEGF-A expression and angiogenesis in human osteosarcoma cells.

### CCL3 promotes VEGF-A expression and angiogenesis through CCR5 receptor

**Figure 3:**
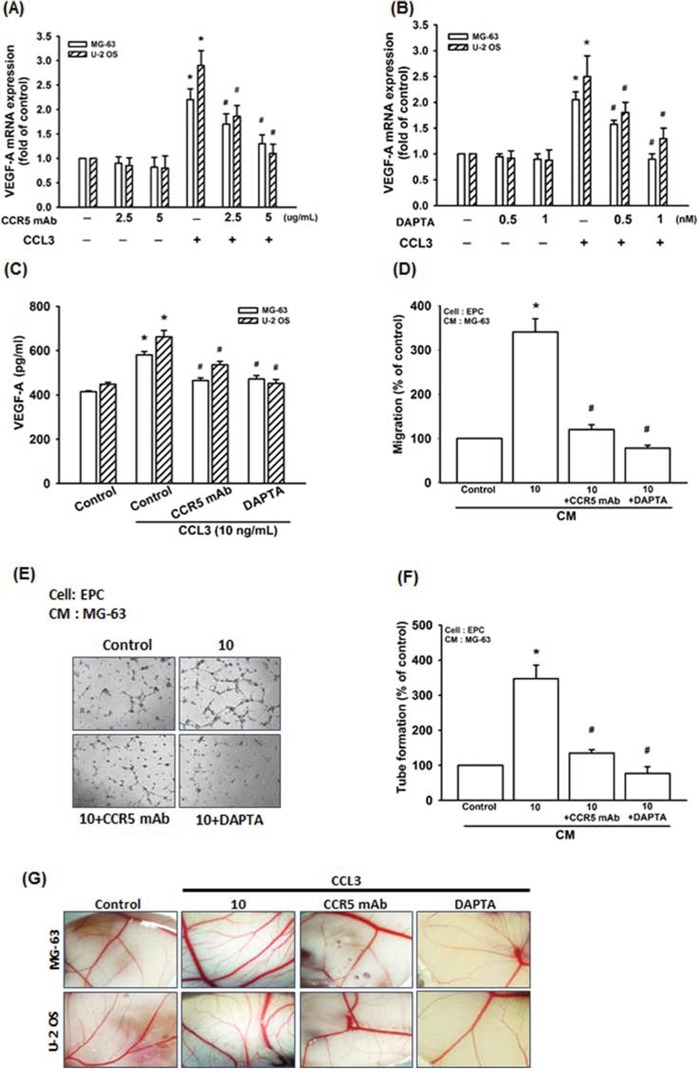
CCL3 promotes VEGF-A expression and angiogenesis through CCR5 receptor Cells were pretreated with CCR5 monoclonal antibody (mAb) (0-5 μg/ml) or the CCR5 antagonist (DAPTA) (0-1 nM) for 30 min, and then treated with CCL3 (10 ng/mL) for 24 h. The mRNA and protein expression of VEGF-A were detected by RT-qPCR **A&B.** and ELISA **C. D&E.** The MG-63 cells culture medium were collected as CM and then applied to EPCs for 24 h. The cell migration and capillary-like structure formation in EPCs was examined by Transwell and tube formation assay. **F.** Quantitative results of EPCs tube formation assay. **G.** Chick embryos were incubated with osteosarcoma CM for 4 days, and then photographed with a stereomicroscope. Each experiment was done in triplicate. Results are expressed as mean ± S.E.M. *P<0.05 compared with control; ^#^*P*<0.05 compared with CCL3-treated group.

CCL3 affects cell functions by binding to CCR5 receptor [13, 30-32]. In our IHC results, we found the expression of CCR5 is higher in patients compared to normal individuals, and the expression profile is also associated with the clinical stage (Fig. 1A). In addition, the CCR5 and VEGF-A are localized in nucleus and cell membrane (Fig. 1A). We examined whether CCL3-induced VEGF-A expression and angiogenesis through CCR5 receptor. Pretreated cells with CCR5 monoclonal antibody (mAb) or the CCR5 antagonist (DAPTA) significantly reduced CCL3-induced VEGF-A mRNA expression in concentration dependent manner (Fig. 3A and 3B). Furthermore, CCR5 mAb or DAPTA prevents CCL3-induced VEGF-A protein expression, cell migration as well as tube formation of EPCs, and new vessels formation in CAM assay (Fig. 3C-3G). These results reveal that CCL3 promotes VEGF-A expression through CCR5, which in turn regulates the angiogenesis within the osteosarcoma microenvironment.

### JNK, ERK, and p38 activation are involved in CCL3-induced VEGF-A expression and angiogenesis

**Figure 4:**
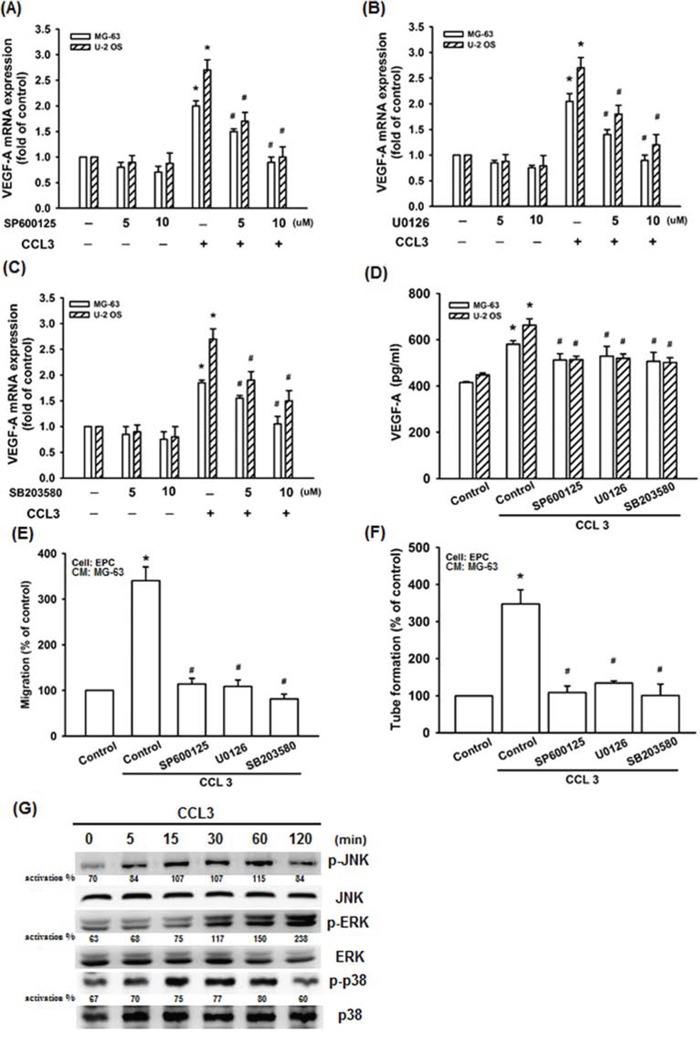
JNK, p38, and ERK activation are involved in CCL3-promoted VEGF-A expression and contributing to angiogenesis Cells were pretreated with SP600125 (JNK inhibitor) (0-10 μM), U0126 (ERK inhibitor) (0-10 μM), or SB203580 (p38 inhibitor) (0-10 μM) for 30 min, and then treated CCL3 (10 ng/mL) for 24 h. The mRNA and protein expression of VEGF-A were detected by RT-qPCR **A-C.** and ELISA **D. E&F.** The culture medium were collected as CM and then applied to EPCs for 24 h. The cell migration and capillary-like structure formation in EPCs was examined by Transwell and tube formation assay. **G.** Cells were incubated with CCL3 (10 ng/mL) for the indicated times, and JNK, p38, and ERK phosphorylation were detected by western blot. The activation % was plotted phosphorylated target/total. Each experiment was done in triplicate. Results are expressed as mean ± S.E.M. *P<0.05 compared with control; ^#^*P*<0.05 compared with CCL3-treated group.

Mitogen-activated protein kinase (MAPK) signaling pathway plays an important role in the regulation of VEGF-A expression level [33]. The MAPK pathway can be grouped into three main families, c-Jun amino-terminal kinase (JNK), extracellular-signal-regulated kinase (ERK), and stress-activated protein kinase (p38) [34]. To further detected whether JNK, ERK, and p38 involved in CCL3-induced VEGF-A expression. We used three inhibitors (SP600125, U0126, and SB203580) which respectively specific for inhibition of JNK, ERK, and p38 [35-38]. As the data shown, incubation of cells with SP600125, U0126, or SB203580 abolished CCL3-induced the expression of VEGF-A mRNA in concentration dependent manner (Fig. 4A-4C). These inhibitors also abolished CCL3-induced VEGF-A protein expression (Fig. 4D). Furthermore, EPC migration and tube formation, which were induced by CCL3-treated osteosarcoma cells CM, were also abolished (Fig. 4E and 4F). In addition, incubation of cells with CCL3 promoted JNK, ERK, and p38 phosphorylation (Fig. 4G). Together, we found that CCL3 induced VEGF-A expression and angiogenesis by activating JNK, ERK, and p38 pathways in human osteosarcoma cells.

### CCL3 promotes VEGF-A expression and angiogenesis by down-regulating miR-374b expression

**Figure 5:**
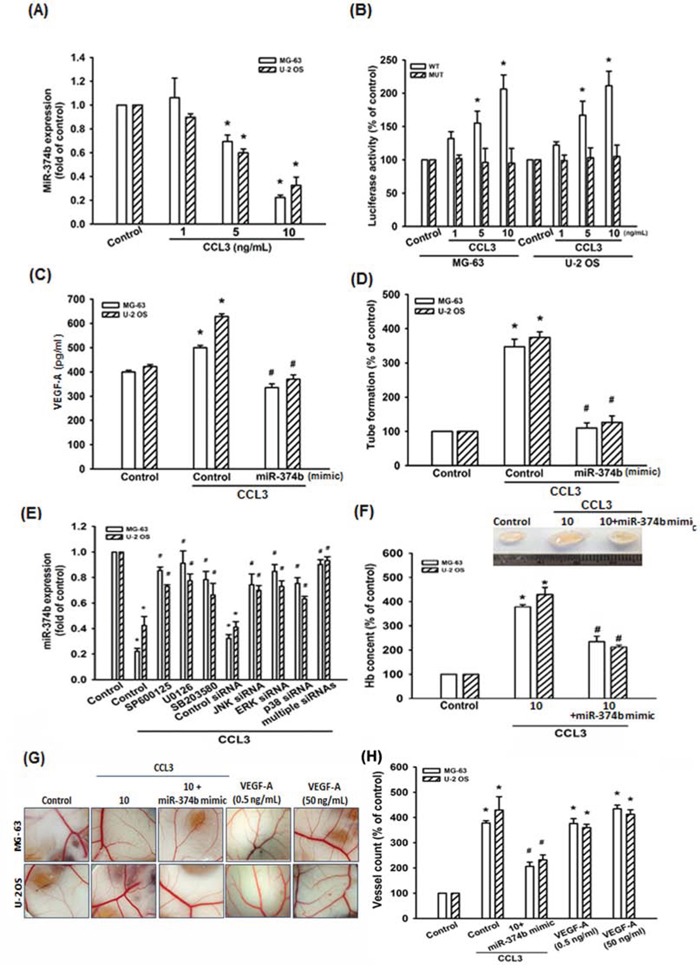
CCL3 promotes VEGF-A expression and angiogenesis by downregulating miR-374b **A.** Cells were treated with CCL3 (1-10 ng/mL) for 24 h, and miR-374b expression was detected by RT-qPCR. **B.** Cells were transfected with wild-type (pmirGLO-VEGF-A-WT; WT) or mutant (pmirGLO-VEGF-A-MUT; MUT) 3′ UTR reporter assay plasmid for 24 h, and then incubated with CCL3 (1-10 ng/mL), the relative luciferase activity was measured. **C.** Cells were transfected with miR-374b mimic for 24 h, and then incubated with CCL3 (10 ng/mL), and the VEGF-A expression was detected by ELISA kit. **D.** The culture medium were collected as CM and then applied to EPCs for 24 h. The capillary-like structure formation in EPCs was examined by tube formation assay. **E.** Cells were pre-treated with SP600125, U0126, SB203580 for 30 min or pre-transferated with JNK-, ERK-, p38-, or multiple (combining JNK-, ERK-, and p38-) siRNA for 24 h, then treated cells with CCL3 (10 ng/mL) for 24 h. The miR-374b expression was detected by RT-qPCR. **F.** Mice were injected subcutaneously with Matrigel mixed with osteosarcoma CM for 7 days, and then the plugs were excised from mice, photographed and quantified the hemoglobin content. **G&H.** Chick embryos were incubated with osteosarcoma CM or VEGF-A (0.5 and 50 ng/mL) for 4 days, and then photographed with a stereomicroscope. Each experiment was done in triplicate. Results are expressed as mean ± S.E.M. *P<0.05 compared with control; ^#^*P*<0.05 compared with CCL3-treated group.

MiRNAs are important regulators in tumor angiogenesis, which makes them promising therapeutic targets [39]. Therefore, we next searched for possible miRNAs responsible for regulating VEGF-A expression using open sourced software (www.TargetScan.org and www.microrna.org). We ranked the top 24 miRNAs that harboring the binding sites of VEGF-A. After stimulation with CCL3, we found miR-374b was the most down-regulated in response to CCL3 (Supplementary Table S1), which is also conserved across species (Supplementary Fig. S3). Besides, we also detected the expression of miR-374b in Control- and CCL3-shRNA stabled cells. We found that the expression of miR-374b was elevated >3-folds in CCL3-shRNA than Control-shRNA cells (Supplementary Fig. S4). Direct application of CCL3 in MG-63 and U-2 OS cells for 24 h reduced miR-374b expression dose-dependently (Fig. 5A). To determine whether CCL3-reduced miR-374b regulates VEGF-A expression by binding to VEGF-A 3′ UTR, we constructed luciferase reporter vectors harboring the wild-type (pmirGLO-VEGF-A-WT; WT) or mutant (pmirGLO-VEGF-A-MUT; MUT) 3′ UTR of the VEGF-A, which has miR-374b binding site (Supplementary Fig. S5), and transfected into MG-63 and U-2 OS cells. As shown in Fig. 5B, CCL3 promoted luciferase activity was increased in wild-type but not in mutant. MiR-374b mimic was used to determine the effect of CCL3-reduced miR-374b in VEGF-A expression and angiogenesis (the transfection efficiency was shown in Supplement Fig. S7). We found that transfection with miR-374b mimic abolished CCL3-induced VEGF-A expression and tube formation in EPCs (Fig. 5C and 5D). Besides, cells pre-treated with SP600125, U0126, and SB203580 or pre-transfected with JNK-, ERK-, and p38-siRNA reversed CCL3-reduced miR-374b expression (Fig. 5E and Supplementary Fig. S6). Particularly, combining multiple (JNK-, ERK-, and p38-) siRNAs returned miR-374b expression in osteosarcoma cells (Fig. 5E). These data suggests that CCL3 induces VEGF-A expression and angiogenesis by down-regulating miR-374b through JNK, p38, and ERK pathways.

Matrigel plugs assay was performed to confirm CCL3-mediated angiogenesis by down-regulation of miR-374b expression *in vivo*. The results showed that Matrigel mixed with CM from CCL3-treated cells increased microvessel formation. In contrast, the CM from miR-374b mimic treated cells abolished CCL3-mediated angiogenesis (Fig. 5F-5G). Quantification of the level of angiogenesis by examining hemoglobin content revealed that CCL3 enhanced angiogenesis *in vivo* (Fig. 5F). We also used CAM assay to confirm the results from the Matrigel model. It was found that CM from CCL3-treated group promoted angiogenesis in CAM model, as shown in Fig. 5G-5H. Moreover, transfection of cells with miR-374b mimic abolished CCL3-mediated angiogenesis in the CAM (VEGF-A was used as positive control). These results demonstrated that CCL3 promotes angiogenesis through down-regulation of miR-374b expression.

### CCL3 increases angiogenesis in mice xenograft model

**Figure 6:**
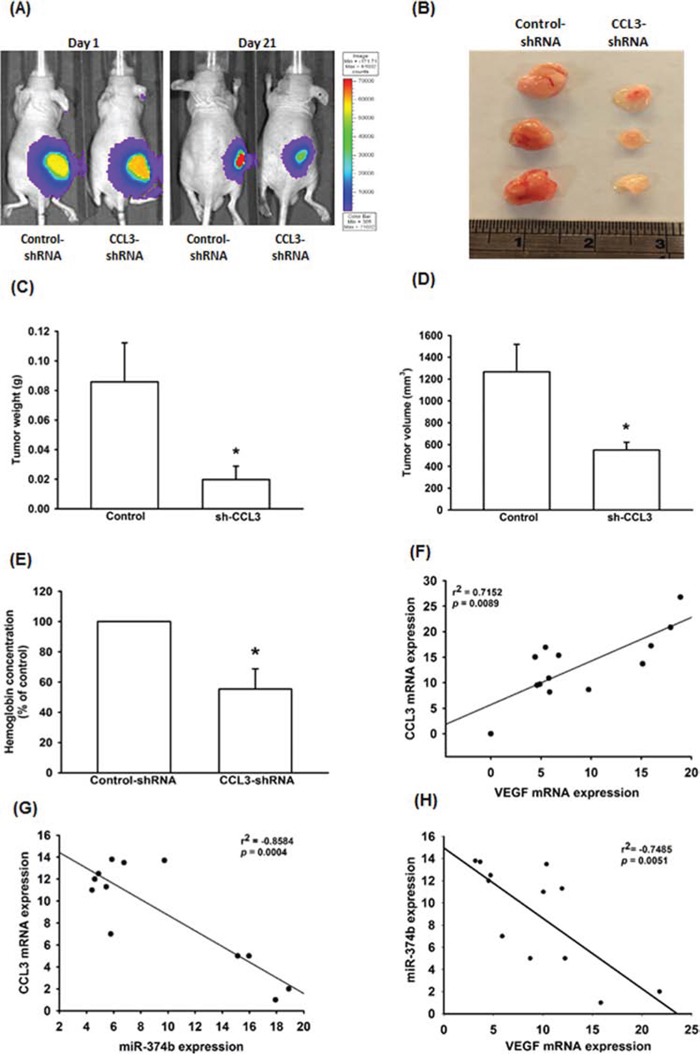
CCL3 has importance effect on in vivo angiogenesis and clinical significance **A.** U-2 OS/Control-shRNA or U-2 OS/CCL3-shRNA cells were subcutaneously injected into the right flank, and tumors were monitored by bioluminescence imaging at 1 and 21 days. After 21 days, the mice were sacrificed and the tumors excised. The tumors were photographed with a microscope **B.** measured weight **C.** and volume **D.** and quantified the hemoglobin levels **E.** The mRNA expression of CCL3, VEGF-A and miR-374b in osteosarcoma patients was examined by RT-qPCR. The correlation between CCL3/VEGF-A **F.** CCL3/miR-374b **G.** and VEGF-A/miR-374b **H.** Each experiment was done in triplicate. Results are expressed as mean ± S.E.M. *P<0.05 compared with control.

CCL3-mediated angiogenesis was further demonstrated by *in vivo* mice xenograft model. As shown in Fig. 6A-6D, knockdown of CCL3 profoundly suppressed tumor growth in mice. We also evaluated the level of angiogenesis by measured the hemoglobin concentration in tumor specimens. The results show that knockdown of CCL3 decrease 40% of hemoglobin concentration in tumor (Fig. 6E). Overall, these results suggest that CCL3 promotes angiogenesis and tumor growth *in vivo*.

### Clinical importance of CCL3, VEGF-A, and miR-374b in osteosarcoma patients

To extend our analysis to clinical osteosarcoma, we collected 13 osteosarcoma samples from patients from the China Medical University Hospital. The mRNAs were isolated from the samples for investigating the clinical importance of CCL3, VEGF-A, and miR-374b. We found that the mRNA expression of CCL3 has positive correlation with VEGF-A (Fig. 6F), and the miR-374b expression has negative correlation with CCL3 and VEGF-A expression (Fig. 6G and 6H).

## DISCUSSION

**Figure 7:**
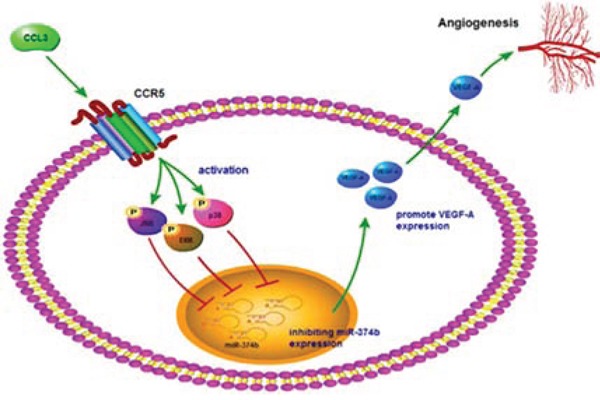
Schematic presentation of the signaling pathways involved in CCL3-induced VEGF-A expression and angiogenesis of osteosarcoma cells CCL3 activates CCR5 receptor and JNK, ERK, or p38 pathway, then down-regulating miR-374b expression, which leads to VEGF-A expression and increases the angiogenesis of human osteosarcoma cells.

Osteosarcoma is the most common primary bone tumor with high morbidity that occurs mainly in children and adolescents. Metastases in patients represents the most common cause of death [3, 40]. Angiogenesis in osteosarcoma microenvironment facilitates tumor progression and metastasis [41]. Previous results demonstrated strong angiogenesis induction by osteosarcoma cells and suggest this process to be a potential therapeutic target for this disease [42]. VEGF-A is a major contributor to angiogenesis [43]. In this study, we found the effect of CCL3 on VEGF-A production in human osteosarcoma, which is responsible for subsequent increased tumor angiogenesis. CCL3 increases angiogenesis and up-regulation of VEGF-A expression by down-regulating miR-374b through JNK, p38, and ERK pathways in human osteosarcoma cells (Fig. 7).

Inappropriate chemokine/receptor expression or regulation has been linked with several diseases, especially those characterized by an excessive cellular infiltrate [44]. There is now overwhelming evidence that chemokines are also involved in the progression of cancer [45]. Overexpression of CCL3 has been observed in B cell-related tumors, including multiple myeloma and chronic lymphocytic leukemia [46, 47]. In our previous study, we demonstrated that CCL3 promotes metastasis of human chondrosarcoma cells [15]. Here, using immunohistochemistry (IHC) analysis, we determined that CCL3 and VEGF-A expressions in osteosarcoma specimens from patients are associated with tumor grade, and are significantly higher compared to normal bone specimens. Furthermore, the mRNA expression of CCL3 has positive correlation with VEGF-A in osteosarcoma patients. We found that CCL3 plays an important role in promoting VEGF-A expression and angiogenesis in human osteosarcoma cells, suggesting that CCL3 may be a novel target for metastasis and angiogenesis of osteosarcoma.

CCL3 regulates several bio-functions by binding to G-protein coupled receptors, CCR1 and CCR5 [11]. The CCR5 receptor is present on the surface of tumor cells and is responsible for CCL3-mediated cell motility and angiogenesis [26, 48]. CCR5 functions as a chemokine receptor in the CC chemokine group, and is expressed by a variety of cells, including lymphocytes, monocytes, neutrophils, and bone marrow progenitor cells [31]. Here we found that CCR5 inhibitor abolished CCL3-induced VEGF-A expression. Furthermore, CCR5 mAb blocked CCL3-mediated VEGF-A production and angiogenesis. These results suggested that CCL3 promotes VEGF-A expression and angiogenesis in human osteosarcoma cells by interacting with CCR5 receptor.

MAPK signaling regulates angiogenesis associated with cytokine secretion in squamous cell carcinoma [49]. Prior study has shown that MAPK is activated after stimulation of CCL3 [50]. In this study we found that stimulation of cells with CCL3 promoted JNK, ERK, and p38 phosphorylation in a time-dependent manner. Pretreatment of cells with JNK, ERK, or p38 inhibitor prevented CCL3-induced VEGF-A expression, EPC migration, and tube formation. Data hint, MAPK pathway plays a key role in CCL3-induced VEGF-A expression and angiogenesis.

A relatively new layer of VEGF-A gene regulation involves miRNA [39]. In osteosarcoma cells, three miRNAs regulates VEGF-A expression including miR-145, miR-29b, and miR-410 [51-53]. However, we found that miR-145, miR-29b and miR-410 only have less regulated by CCL3, and we revealed another miRNA (miR-374b) that could target VEGF-A, and inhibit angiogenesis in human osteosarcoma cells. Data show that CCL3 inhibits miR-374b expression dose-dependently. Transfection with miR-374b mimic halted CCL3-mediated VEGF-A expression. In addition, the expression of miR-374b has negative correlation with CCL3 and VEGF-A in osteosarcoma patients, suggesting miR-374b plays a negative regulator in CCL3-induced VEGF-A expression and angiogenesis. Previous study has revealed that miRNAs are associated with hyper-activation of MAPK signaling [54]. In the present study we found that JNK, ERK, and p38 inhibitor or siRNA reversed CCL3-inhibited miR-374b expression, suggesting that CCL3 reduced miR-374b via JNK, ERK, and p38 pathways.

In conclusion, CCL3 is expressed at high levels in osteosarcoma and promotes VEGF-A expression. Binding of CCL3 to CCR5 receptor inhibits miR-374b expression by activating JNK, ERK, and p38 signaling pathways, which subsequently promotes VEGF-A expression and angiogenesis. Thus, CCL3 may be a new molecular therapeutic target in osteosarcoma angiogenesis and metastasis.

## MATERIALS AND METHODS

### Materials

Anti-rabbit and anti-mouse IgG-conjugated horseradish peroxidase, rabbit polyclonal antibody specific for α-tubulin, JNK, p38, and ERK, and mouse monoclonal antibodies specific for CCL3, VEGF-A, CCR5, p-JNK, p-p38, and p-ERK were purchased from Santa Cruz Biotechnology (Santa Cruz, CA). VEGF-A antibody was purchased from Abcam (Cambridge, MA, USA). SP600125, SB203580, U0126 were purchased from Calbiochem (San Diego, CA). ON-TARGETplus siRNAs of JNK (product ID: L00351400), ERK (product ID: L00355500), and p38 (product ID: L00351200) were purchased from Dharmacon Research (Lafayette, CO, USA) (The transfection efficiency was shown in Supplementary Fig. S8). Recombinant human CCL3 and VEGF-A were purchased from PeproTech (Rocky Hill, NJ). Dulbecco's modified Eagle's medium (DMEM), fetal bovine serum (FBS) and all other cell culture reagents were from Gibco-BRL Life Technologies (Grand Island, NY, USA). MiRNome microRNA Profilers QuantiMir™ kit was from System Biosciences (SBI) (Mountain View, CA). MiR-374b mimic was purchased from Invitrogen (Carlsbad, CA). All other chemicals purchased from Sigma-Aldrich (St. Louis, MO).

### Cell culture

The human osteosarcoma cell lines (MG-63; low malignant cell line separated from 14 years Caucasian male and U-2 OS; a high malignant cell line separated from 15 years Caucasian female) were purchased from the Bioresource Collection and Research Center (BCRC) (Hsinchu, Taiwan). Short tandem repeat profiles were examined by BCRC before and after our study (July, 2014) to ensure the quality and integrity of these two cell lines. MG-63 were maintained in Dulbecco`s Modified Eagle Medium (DMEM) and U-2 OS cells were maintained in McCoy's 5A medium, which was supplemented with 20 mM HEPES and 10% heat-inactivated FCS, 2 mM glutamine, penicillin (100 U/ml), streptomycin (100 μg/ml) and 10% FBS at 37°C with 5% CO_2_.

### Endothelial progenitor cell (EPC) culture

Protocol for EPC culture was approved by the Institutional Review Board of Mackay Medical College, New Taipei City, Taiwan (reference number P1000002). The preparation of EPC was described before [9]. EPCs were cultured in MV2 complete medium that contained MV2 basal medium and growth supplement (PromoCell, Heidelberg, Germany) and was also supplemented with 20% defined FBS (HyClone, Logan, UT, USA). Cultures were seeded on 1% gelatin-coated plasticware and maintained at 37°C in a humidified 5% CO_2_ atmosphere.

### Construction of stable expression CCL3 shRNA cell line

The CCL3 shRNA was purchased from the National RNAi Core Facility (NRC) (Academia Sinica, Taipei, Taiwan). The oligo sequence of CCL3 shRNA was 5′-CCGGCCGGCAGATTCCACAGAATTTCTCGAGAAA TTCTGTGGAATCTGCCGGTTTTTG-3′. Cells were seeded in 6 cm plates 24 h. Remove growth media and replace with fresh media containing polybrene (5 μg/ml). Infect cells by adding the CCL3 shRNA lentiviral particles to the culture. Swirl the plate to mix and incubate overnight. Remove media and replace with 5 mL fresh growth media containing with 2 μg/ml puromycin to select for stable transfectants. Single clones were picked, the ectopic expression of the gene of interest was verified using Western blot analysis.

### Patients and specimen preparation

The study protocol was approved by the Institutional Review Board of China Medical University Hospital. All patients gave written consent before enrollment. The tumor tissue specimens were collected from patients who were diagnosed with osteosarcoma and had undergone surgical resection at China Medical University Hospital.

### Preparation of conditioned medium (CM)

Osteosarcoma cells were either treated with CCL3 alone for 24 h or were pretreated with pharmacological inhibitors, siRNA, or miR-374b mimic followed by stimulation with CCL3 for 24 h. The cells were washed and transferred to serum-free medium after treatment. Conditioned medium (CM) was then collected 2 days after the change of medium and stored at −80°C until use. In the provided concentration, these inhibitors, siRNA, or miR-374b mimic did not significantly affect cell viability.

### EPC migration assay

Cell migration assay was performed using Transwell chambers with 8.0 μm pore size (Coring, Coring, NY). EPCs (1 × 10^4^ cells/well) were seeded onto the upper chamber with MV2 complete medium, and then incubated in the bottom chamber with 50% MV2 complete medium and 50% osteosarcoma cell CM. The plates were incubated for 24 h at 37°C in 5% CO_2_, and then the cells were fixed in 4% formaldehyde solution for 15 min and stained with 0.05% crystal violet in phosphate-buffered saline (PBS) for 15 min. Cells on the upper side of the filters were removed with cotton-tipped swabs, and the filters were washed with PBS. Cell migration was quantified by counting the number of stained cells in 10 random fields using an inverted phase contrast microscope and then photographed.

### Tube formation assay

Matrigel (BD Biosciences, Bedford, MA) was dissolved at 4°C overnight, and 48-well plates were prepared with 150 μl Matrigel in each well, and then incubating at 37°C for 30 min. After gel formation, EPCs (1×10^4^ cells) were seeded per well on the layer of polymerized Matrigel in cultured media containing 50% MV2 complete medium and 50% osteosarcoma cell CM, followed by incubation for 16 h at 37°C. Then, the EPC tube formation was taken with the inverted phase contrast microscope. Tube branches and total tube length were calculated using MacBiophotonics Image J software.

### Immunohistochemical (IHC) staining

Human osteosarcoma tissue array was purchased from US Biomax Inc., (MD, USA) and sections were deparaffinized with xylene and rehydrated by adding ethanol. Endogenous peroxidase activity was blocked with 3% hydrogen peroxide in methanol for 10 min. Heat-induced antigen retrieval was carried out for all sections in 0.01 M sodium citrate buffer, pH 6 at 95°C for 25 min. Human CCL3, CCR5, and VEGF-A antibodies were applied at a dilution of 1:300 and incubated at 4°C overnight. The antibody-binding signal was detected using the NovoLink Polymer Detection System (Leica Microsystems) and visualized with the diaminobenzidine reaction. The sections were counterstained with hematoxylin. All sections were evaluated with one blind and independently by pathologist (Dr. I-Ping Chiang). The immunohistochemistry results were scored by taking into account the percentage of positive detection and intensity of the staining. The percentage of positive *(p)* score was given as follows: 0, no staining; 1^+^, <10% of cells stained; 2^+^, 10-25% of cells stained; 3^+^, 25-50% of cells stained; 4^+^, 50-75% of cells stained; 5^+^, >75% of cells stained. Simultaneously, the staining intensity *(I)* was estimated and expressed as weak, moderate, or strong (score 0.1, 0.5, or 1). Results are scored by multiplying the percentage of positive cells *(P)* by the intensity *(I)*. Formula: Q = *P* × *I*.

### ELISA assay

Human osteosarcoma cells were cultured in 6-well plates. After reaching confluence, cells were changed to serum-free medium. Cells were then treated with CCL3 alone for 24 h, or pretreated with pharmacological inhibitors, or transfected with specific siRNA, or miR-374b mimic followed by stimulation with CCL3 for 24 h. After treatment, the medium was removed and stored at −80°C. Then, VEGF-A in the medium was determined using VEGF-A ELISA kit (PeproTech, Rocky Hill, NJ) according to the manufacturer's protocol.

### Cell viability assay (MTT assay)

Cell viability was determined by the MTT assay. Control-shRNA or CCL3-shRNA cells were plated in 96-well plates at a concentration of 2,000 cells per well. After 0, 6, 12, 24, 48, 72 h, cells were washed with PBS. Next, 0.5 mg/ml of MTT solution was added to each well and incubated at 37°C for 30 min. To dissolve formazan crystals, culture medium was replaced with an equal volume of DMSO. After the mixture was shaken at room temperature for 10 min, absorbance of each well was determined at 550 nm using a microplate reader (Bio-Tek, Winooski, VT, USA).

### 3′ UTR reporter assay plasmid

The 3′ UTR regions of human VEGF-A gene including miR-374b binding site was amplified by PCR. Amplification conditions were 95°C for 3 min followed by 40 cycles of 95°C for 30 s and 62°C for 40 s using primers sequences flanked by NheI and XhoI sites: (forward) 5′-GACACACCCACCCACATACA-3′ and (reverse) 5′-TCTCCTCCTCTTCCCTGTCA-3′. The 3′ UTR were cloned in pmir-GLO vector (Promega, Madison, WI, USA) downstream of the reporter gene. The predicted VEGF-A binding sites, identified by the miRDB (http://mirdb.org/miRDB). Mutant plasmids were generated using a QuickChange Site-Directed Mutagenesis kit (Stratagene, Cedar Creek, TX, USA).

### Dual-luciferase reporter assay

To perform the luciferase reporter assay, MG-63 or U-2 OS cells ( 2 × 10^5^) were seeded in a 24-well plate and then transfected with 1 μg of either the wild type (pmirGLO-VEGF-A-WT; WT) or mutant (pmirGLO-VEGF-A-MUT; MUT) report vector using Lipofectamine™ 2000. After 24 hours transfection, cells were collected for analysis using a Dual-Luciferase Reporter Assay Kit (Promega, USA), in accordance with the manufacturer's instructions. The relative luciferase activity was calculated by the ratio of luciferase/renilla activity, and normalized to that of the control cells. Transfection was done in duplicates and experiments were repeated three times.

### Western blot analysis

Protein concentration was determined using the Thermo Scientific Pierce BCA Protein Assay Kit (Thermo Fisher Scientific Inc., USA). Proteins were resolved on SDS-PAGE and transferred to immobilon polyvinyl difluoride (PVDF) membranes. The blots were blocked with 4% BSA for 1 h at room temperature and incubated with primary antibodies for 1 h at room temperature. After three washes in Tris-buffered saline with 0.05% Tween 20 (TBS-Tween), the blots were subsequently incubated with a donkey anti-rabbit or anti-mouse peroxidase-conjugated secondary antibody for 1 h at room temperature. The blots were visualized by enhanced chemiluminescence using Kodak X-OMAT LS film (Eastman Kodak, Rochester, NY). Quantitative data were obtained using a computing densitometer and ImageQuant software (Molecular Dynamics, Sunnyvale, CA).

### Quantitative real-time PCR

Total RNA was extracted from osteosarcoma cells using a TRIzol kit (MDBio Inc., Taipei, Taiwan). The reverse transcription reaction was performed using 2 μg of total RNA that was reverse transcribed into cDNA using an oligo(dT) primer. Quantitative real-time polymerase chain reaction (q-PCR) analysis was carried out using TaqMan® one-step PCR Master Mix (Applied Biosystems, Foster City, CA, USA). Total complementary DNA (100 ng/25 μL reaction) was mixed with sequence-specific primers and TaqMan® probes according to the manufacturer's instructions. Sequences for all target gene primers and probes were purchased commercially (β-actin was used as the internal control) (Applied Biosystems). Q-PCR assays were carried out in triplicate using a StepOnePlus sequence detection system. The cycling conditions were 10 min of polymerase activation at 95°C, followed by 40 cycles at 95°C for 15 s and 60°C for 60 s.

For miRNAs detection, reverse transcription was performed using Mir-X™ miRNA First-Strand Synthesis and SYBR® qRT-PCR (Clontech Laboratories, Inc., CA, USA). U6 snRNA levels were used for normalization. The specific forward primer of miR-374b was as follows: 5′-ATATAATACAACCTGCTAAGTG-3′. Forward and reverse primers for U6 were 5′-CTCGCTTCGGCAGCACATATACT A-3′and 5′-ACGAATTTGCGTGTCATCCTTGCG-3′. The threshold was set above the non-template control background and within the linear phase of target gene amplification to calculate the cycle number at which the transcript was detected (denoted as CT).

### Chick chorioallantoic membrane assay

Fertilized chicken eggs were incubated at 38°C in an 80% humidified atmosphere. On day 7, CM from control-shRNA or CCL3-shRNA cells (2×10^4^ cells) deposited in the center of the chorioallantoic. CAM results were analyzed on the fourth day. Chorioallantoid membranes were collected for microscopy and photographic documentation. Angiogenesis was quantified by counting the number of blood vessel branch; at least 10 viable embryos were tested for each treatment. All animal works were done in accordance with a protocol approved by the China Medical University (Taichung, Taiwan) Institutional Animal Care and Use Committee.

### *In vivo* Matrigel plug assay

Osteosarcoma cells were transfected with miRNA control or miR-374b mimic for 24 h and then treated with CCL3 for 24 h. CM was then collected. Thirty male BALB/c nude mice (4 weeks of age; purchased from National Laboratory Animal Center, Taipei, Taiwan) were used and randomized into three groups: PBS (control), control-mimic, and miR-374b-mimic. Each group was subcutaneously injected with 0.2 mL Matrigel containing 0.2 mL osteosarcoma cells CM. On day 10, Matrigel plugs were excised. They were used for measuring the extent of blood vessel formation by hemoglobin assay.

### Mice xenograft assay

Male BALB/c nude mice (5 weeks old) were randomly divided into 2 groups (5 mice per group). U-2 OS/Control-shRNA and U-2 OS/CCL3-shRNA cells (2×10^6^ cells per mouse) were resuspended in serum-free medium with Matrigel at a 1:1 ratio, and then subcutaneously injected into the right flank of each animal. Mice body weights were recorded twice weekly. Tumor volume was monitored by the Xenogen IVIS system and images were captured 10 min after D-luciferin injection with a 60-s exposure using a CCD camera. After 21 days, mice were euthanized by subjecting them to CO_2_ inhalation and the tumor volume was calculated using the formula: V = (LW2)π/6, where V is the volume (mm^3^), L is the largest diameter (mm); and W is the smallest diameter (mm).

### Hemoglobin assay

All the Matrigel plugs and tumors were processed for measuring blood vessel formation. Briefly, the amount concentration of hemoglobin in the vessels that have invaded the Matrigel was determined with Drabkin's reagent (Sigma-Aldrich) according to the manufacturer instructions. Take the same weight of plugs or tumors. Homogenized in 1 mL of RIPA lysis buffer and then centrifuged at 1000 rpm., 20 μL of supernatants were added to 100 μL of Darkin's solution. The mixture was allowed to stand for 30 min at room temperature, and then readings were taken at 540 nm in a spectrophotometer.

### Statistics

Data are expressed as the mean ± standard error. The differences between groups were analyzed using the Student's *t*-test of variance. The difference was considered significant if the *p* value was less than 0.05.

## SUPPLEMENTARY FIGURES AND TABLES


