# Supplementary material for: CCL3 promotes angiogenesis by dysregulation of miR-374b/ VEGF-A axis in human osteosarcoma cells
Source: Oncotarget. 2015 Dec 21;7(4):4310–25. doi: 10.18632/oncotarget.6708 (PMC4826207; doi:10.18632/oncotarget.6708)
Supplement: Supplementary file 1 [file oncotarget-07-4310-s001.pdf]

SUPPLEMENTARY FIGURES AND TABLE

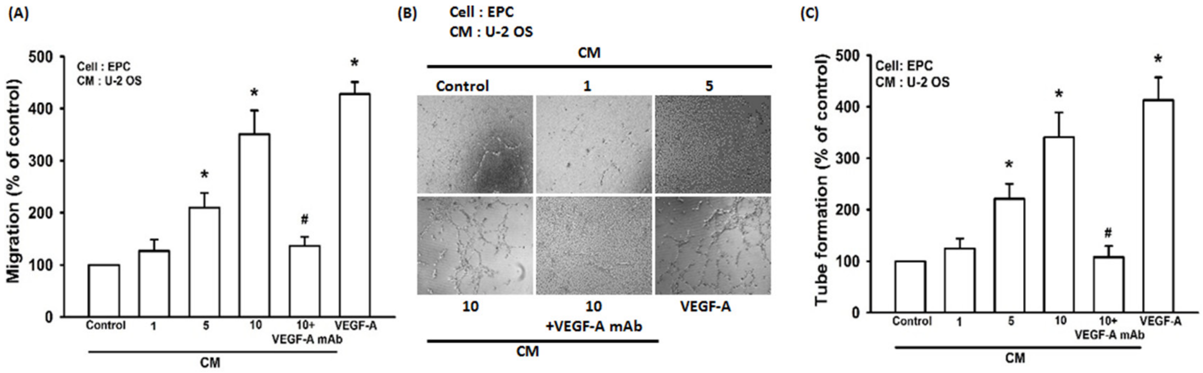

Supplementary Figure S1: CCL3 enhances angiogenesis by increasing VEGF-A expression in human osteosarcoma cells. U-2 OS cells were pre-treated for 30 min with VEGF-A antibody (20 ng/mL), followed by stimulation with CCL3 (10 ng/mL) or incubated with CCL3 (1-10 ng/mL) for 24 h. The culture medium was collected as CM and then applied to EPCs for 24 h. The cell migration and capillary-like structure formation in EPCs was examined by Transwell and tube formation assay. Each experiment was done in triplicate. Results are expressed as mean ± S.E.M. *P<0.05 compared with control; #P<0.05 compared with CCL3-treated group.

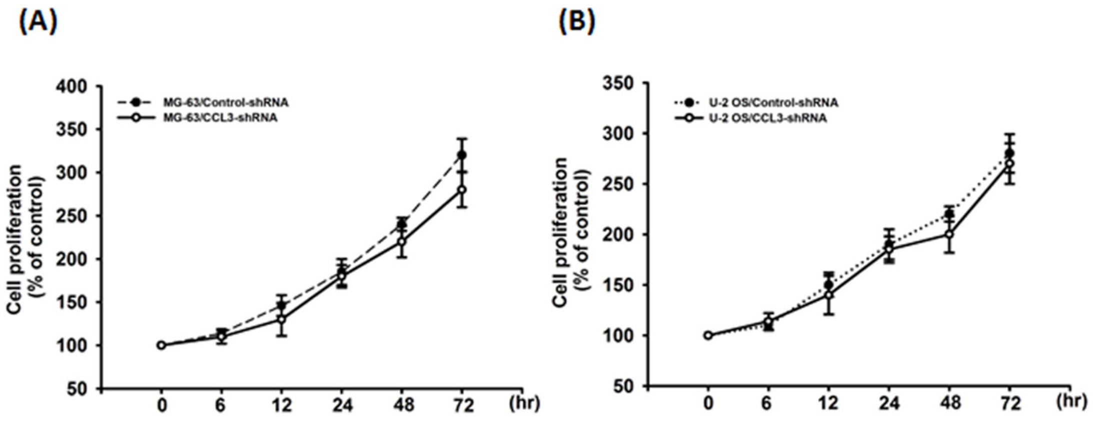

Supplementary Figure S2: Cell viability of stable expression of Control-shRNA or CCL3-shRNA. Cells were plated in 96-well plate at a concentration of 2,000 cells per well. After 0, 6, 12, 24, 48, 72 h, cells were collected and the viability was analyzed by using the MTT assay.

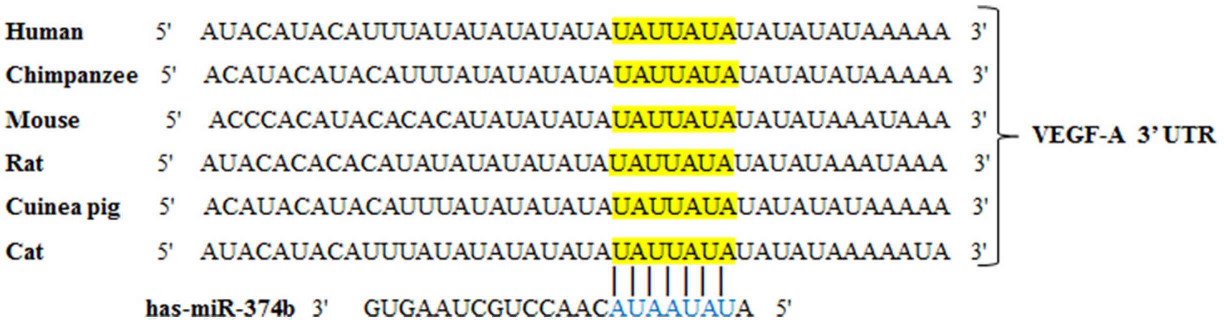

Supplementary Figure S3: Alignment of miR-374b potential target sites across different species. MiR-374b species-specifically target on VEGF-A 3' UTR in different species. Note that the miR-374b binding seed region is conserved across species.

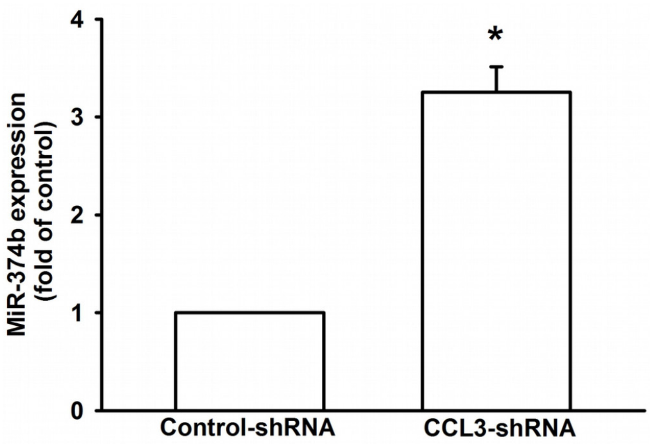

Supplementary Figure S4: The expression of miR-374b in Control- and CCL3-shRNA stabled cells. RT-qPCR analysis of miR-374b expression in Control- and CCL3-shRNA stabled cells, and found the miR-374b expression was >3-folds in CCL3-shRNA than Control-shRNA cells.

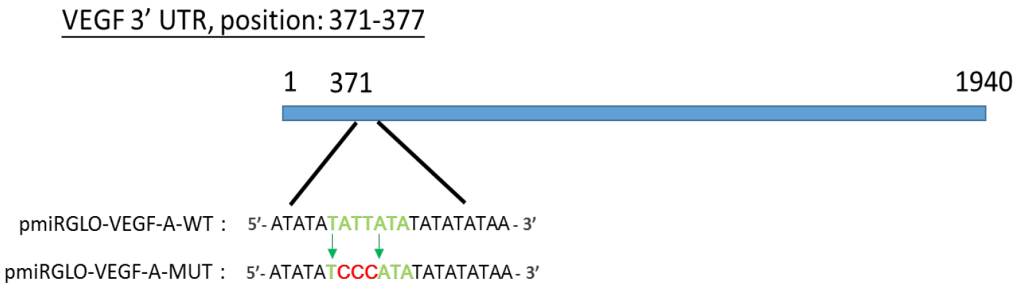

Supplementary Figure S5: Schematic illustrations of the pmirGLO Dual-Luciferase reporter construct for examining the effect of miR-374b on VEGF-A 3'UTR. The full-length sequence of the VEGF-A 3'UTR is located at position 1687 of VEGF-A mRNA (NM_003376). The miR-374b seed location in VEGF-A 3'UTR is 371 to 377. The fragment that contains the predicted miR-374b binding site was cloned into dual-luciferase report plasmid. The construct with the VEGF-A 3'UTR mutated at the predicted miR-374b binding site are also depicted.

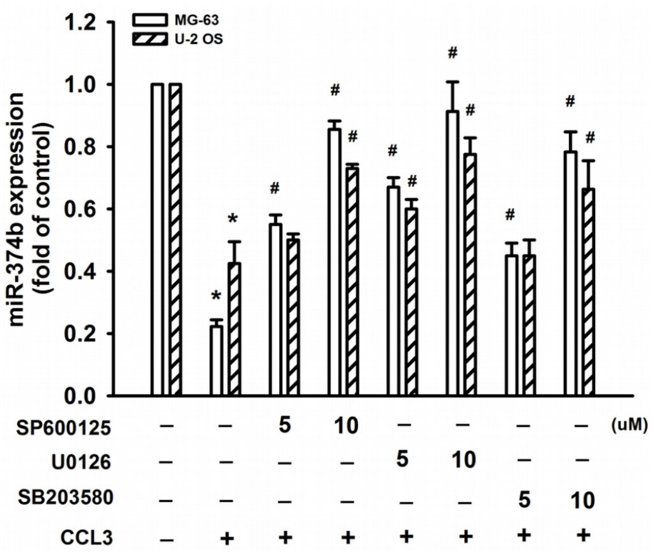

Supplementary Figure S6: MAPK pathway is involved in CCL3-inhibited miR-374b expression. Cells were pre-treated with SP600125, U0126, SB203580 for 30 min, then treated cells with CCL3 (10 ng/mL) for 24 h. The miR-374b expression was detected by RT-qPCR.

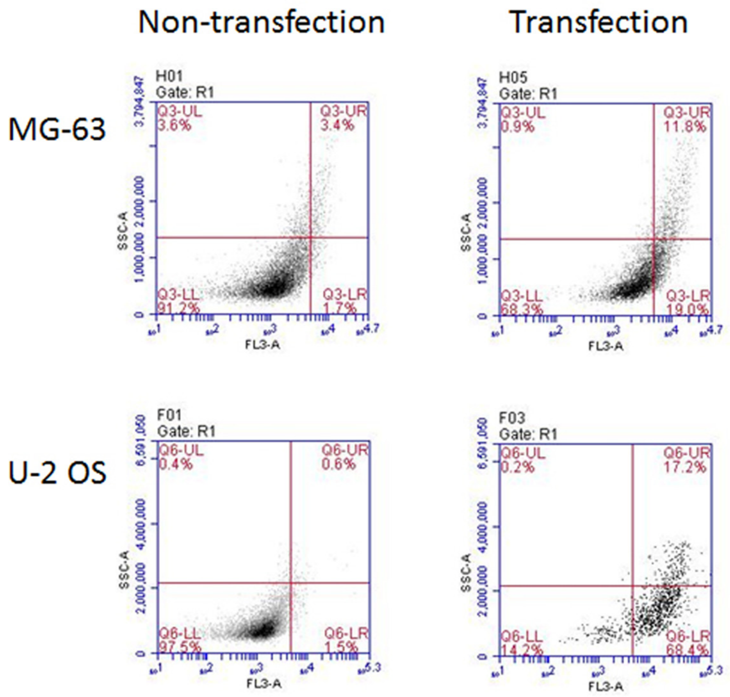

Supplementary Figure S7: Dye-labeled miRIDIAN mimic transfection controls allow for qualitative evaluation of transfection. Transfection efficiency for the microRNA mimic was assessed using Dy574-labeled miRNA negative control mimic precursor Dharmacon Research (Lafayette, CO, USA). The transfection effect was evaluated using flow cytometry, and the efficiency of MG-63 and U-2 OS cells were 40.35% ± 10% and 88.26% ± 9% (mean ± SD) respectively.

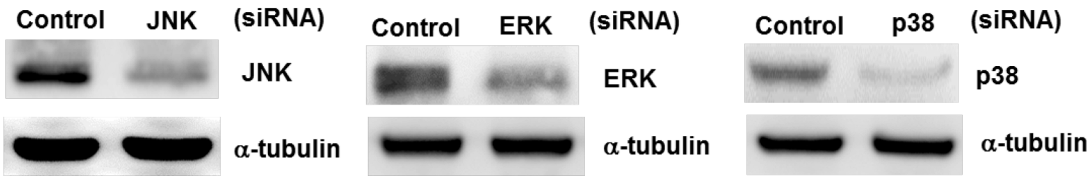

Supplementary Figure S8: Protein expression of JNK, ERK, and p38 after siRNA transfection. Cells were seeded in a 6-well plate and then transfected with 100 (nM) of siRNA. After 24 h, cells were collected and the protein expression was detected by western blot.

Supplementary Table S1: Representative miRNA expression after CCL3 stimulation

miRNA name	Fold	miRNA name	Fold
miR-374b	0.113	miR-29c	0.596
miR-127-5p	0.216	miR-206	0.632
miR-484	0.432	miR-29b	0.646
miR-511	0.432	miR-145	0.877
miR-655	0.432	miR-410	0.987
miR-519d	0.433	miR-200b	1.101
miR-150	0.465	miR-578	1.238
miR-93	0.465	miR-200c	1.258
miR-134	0.465	miR-300	1.467
miR-106a	0.495	miR-24	2.127
miR-494	0.499	miR-16	2.656
miR-29a	0.539	miR-452	2.994

RT-qPCR analysis of < 3-fold expression of miRNAs in CCL3 stimulated MG-63 cells.
